# Project PRIME (Psychosocial Response to International Medical Electives): Results from Medical Trainees

**DOI:** 10.5334/aogh.4627

**Published:** 2025-03-10

**Authors:** Nicole E St Clair, Kristina Devi Singh-Verdeflor, Vanessa McFadden, Elizabeth Groothuis, Stephanie Lauden, Megan S McHenry, Stephen Merry, Stephen Warrick, Samantha L Wilson, James H Conway

**Affiliations:** 1 Associate Professor of Pediatrics at the University of Wisconsin School of Medicine and Public Health and Director of their Pediatric Residency Global Child Health Training Program, USA; 2 Researcher at the University of Wisconsin School of Medicine and Public Health, USA; 3 Associate Professor of Pediatrics at the Medical College of Wisconsin and Associate Section Chief for Hospital Medicine, USA; 4 Assistant Professor of Pediatrics at Northwestern University Feinberg School of Medicine and Assistant Program Director of McGaw Medical Center’s Global Health Clinical Scholars Program, USA; 5 Chief Medical Officer, Lower Lights Health, Columbus, OH, USA; 6 Associate Professor of Pediatrics within the Ryan White Center for Pediatric Infectious Disease and Global Health at Indiana University School of Medicine, USA; 7 Associate Professor of Family Medicine at the Mayo Clinic Alix School of Medicine and Chair of the Mayo International Health Program, USA; 8 Volunteer Assistant Professor of Clinical Pediatrics, Cincinnati Children’s Hospital, University of Cincinnati College of Medicine, USA; 9 Professor of Pediatrics within the Division of Pediatric Psychology and Developmental Medicine, Medical College of Wisconsin, USA; 10 Professor of Pediatrics at the University of Wisconsin School of Medicine and Public Health, the Pediatric Infectious Diseases Fellowship Program Director, and the Director of the Office of Global Health, USA

**Keywords:** global health, global health electives, medical students, medical trainees, culture shock

## Abstract

*Background:* Participation in global health (GH) training experiences is common for US medical trainees (students, residents, and fellows). However, little is known about their experience of “culture shock” (CS), which frequently occurs during these transformative cross‑cultural immersions.

*Objectives:* The objectives of this study include: (1) quantitatively measure medical trainee psychosocial responses to short‑term GH electives, (2) identify factors that influence their CS experiences, and (3) determine if the stage‑based CS conceptual framework applies to medical trainees.

*Methods:* Undergraduate and graduate medical education trainees (UME and GME) who participated in short‑term GH electives between 2016 and 2020 were recruited across nine US institutions. Using a longitudinal survey method, we gathered data predeparture (demographics, resilience, perceived stress (PS), and CS assessments), every 5 days during the elective (CS, PS assessments, and training site conditions), and 30 days postreturn (perceptions of CS experiences). Analyses included summary statistics, linear regressions, and a linear mixed effects model (LMM).

*Findings:* 252 trainees were enrolled, with 140 (56%) included in the LMM. The primary outcome was a culture shock profile (CSP) score, with 96% reporting CS. The only trainee‑specific factor that significantly increased CSP score was trainee type (UME > GME (+22%)). Several GH elective site‑specific factors significantly influenced CSP score (e.g., support network [−10%], role clarity [−11%], and overwhelmed by medical needs [+10%]). CS experiences were variable and did not progress in predictable, stage‑based fashions, which is discordant from common CS descriptions.

*Conclusions:* Culture shock was a near‑universal, diverse experience during GH electives. On‑site training conditions and elective site host factors influenced CS more than trainee factors in this prepandemic cohort. Further research is required to (1) determine the optimal CS “balance” (i.e., promoting transformative learning while mitigating negative professional and personal impacts), (2) offer insight into harmful CS thresholds, (3) identify host perspectives, and (4) inform best practices for GH electives and global partnerships.

## Background

Transformative. Overwhelming. Impactful. These are just a few of the terms that medical trainees (students, residents, and fellows) have used to describe global health (GH) electives, defined here as short‑term training experiences in culturally and geographically distinct settings, often with resource constraints when compared with one’s home institution. Medical trainees in the USA commonly pursue these experiences during their education, ranging from observational to hands‑on work in partnership with local providers: 57% of medical students recently surveyed across 12 US medical schools participated in or intend to participate in a GH elective, and 46% of students stated that GH opportunities would impact their ranking of residency programs [1].

It is anticipated that medical trainees experience emotional highs and lows during these electives, with variable levels of intensity and stress [2]. Common symptoms attributed to cross‑cultural immersion include excitement, irritability, frustration, anxiety, and fatigue, with the negative emotions frequently referred to as “culture shock” (CS). CS is defined in this study as the stress, anxiety, or discomfort a person feels when they are placed in an unfamiliar cultural environment caused by the loss of familiar meanings and cues relating to communication and behavior [3]. These intense emotional responses can produce both significant benefits and harms to the trainees, their hosts, and the host communities. Potential benefits of cross‑cultural immersion to the trainees include fostering transformative learning, professional identity formation, intercultural empathy, cultural humility, and resilience [4–7]. Potential harms include increased stress and exacerbation of preexisting trainee mental health conditions (depression, anxiety, or other), suboptimal trainee performance during stressful periods (including but not limited to decreased professionalism, impaired clinical competency, and increased judgmental behaviors), increased burden to the host institutions and communities required to cope with these negative trainee reactions, and potential negative consequences for training partnerships if visiting trainees cannot maintain professionalism in their interactions with patients and hosts.

**Figure 1:**
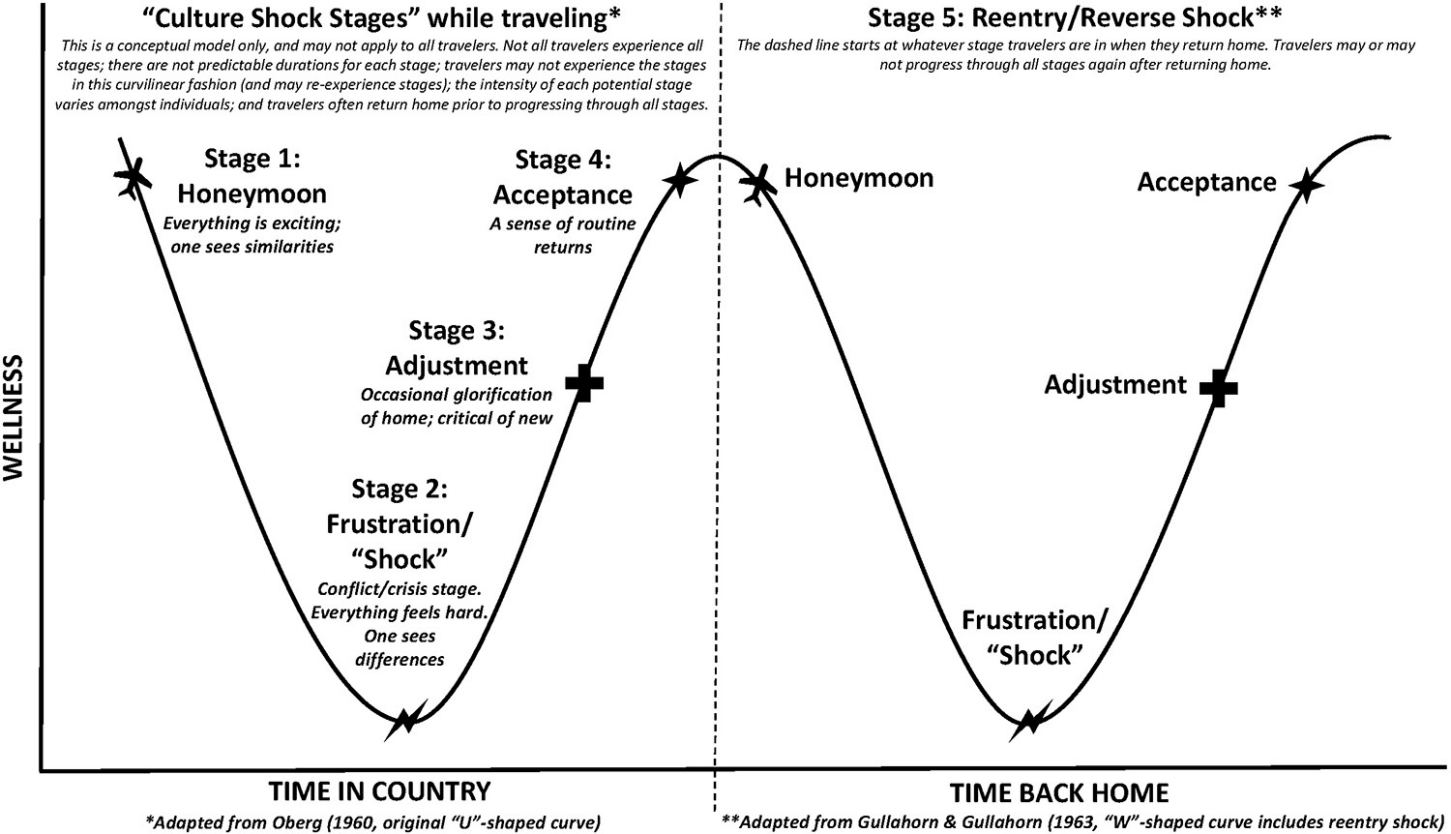
Conceptual framework of culture shock stages used for Project PRIME, adapted from Oberg (1960) and Gullahorn and Gullahorn (1963).

Culture shock was originally described with a “U‑shaped” stage‑based framework by Oberg in 1960 [8], transitioning later to a “W‑shaped” model. It includes the following stages: (1) honeymoon, (2) frustration/“shock”, (3) adjustment, (4) acceptance, and (5) reverse/reentry shock (representing a traveler’s experience upon return to their native culture; Figure 1) [9]. While some researchers have argued that adaptation occurs in a cyclical pattern rather than a stage‑based fashion [7], the stage‑based description is most commonly utilized when describing psychosocial responses to cross‑cultural immersions.

Given widespread interest in GH, and the importance of maximizing benefits and minimizing harm to the trainees, patients, and host communities, it is critical to understand the factors that influence trainee responses to short‑term cross‑cultural clinical immersions [10, 11]. Our study, Project PRIME (Psychosocial Response to International Medical Electives), aims to better understand CS in medical trainees and, in turn, (1) improve educator mentorship pertaining to GH electives (e.g., predeparture advising and preparation, support during the elective, and postreturn debriefing/reintegration); (2) optimize trainee insight regarding anticipated emotional responses to cross‑cultural immersions; (3) foster trainee growth mindsets to promote transformative learning; (4) decrease the burden on host institutions and communities stemming from negative trainee emotional responses; and (5) refine and optimize global training partnerships.

Project PRIME is a multi‑institutional, observational longitudinal survey design study and is the first study to quantitatively measure psychosocial responses to short‑term GH experiences for undergraduate and graduate medical education trainees (UME and GME, respectively), with the following aims: (1) serially assess CS in medical trainees during their GH electives; (2) identify the intrinsic and extrinsic factors that influence the severity of CS experienced by traveling medical trainees; and (3) determine whether medical trainees navigate through CS in the predictable, stage‑based, time‑dependent, curvilinear trajectory described in stage‑based conceptual frameworks.

## Methods

### Study design and setting

**Figure 2:**
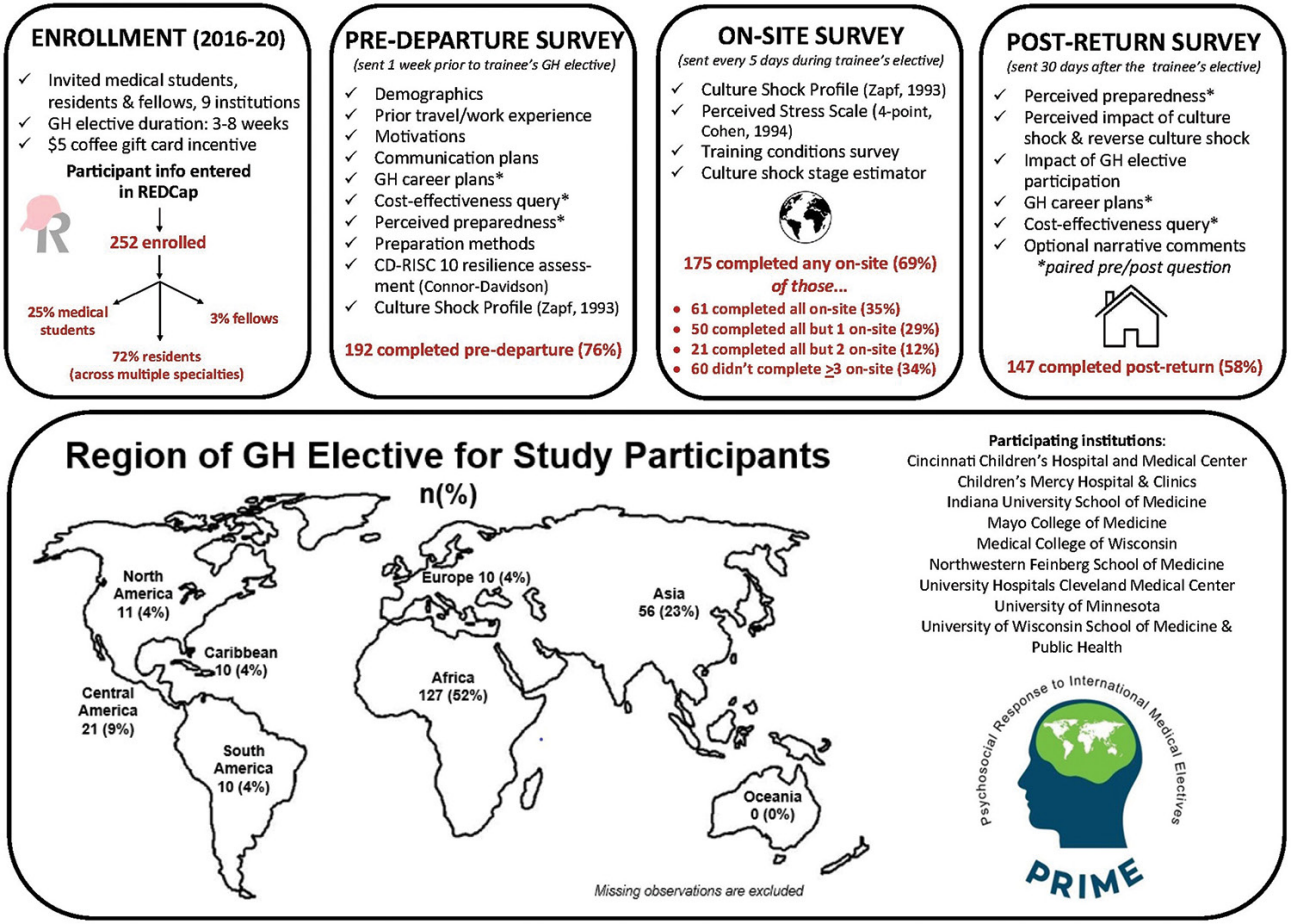
Project PRIME methodology: enrollment, survey distribution, survey completion rates, global health elective regions for study participants, and participating institutions.

This is a multisite longitudinal study of US medical trainees participating in clinical GH electives of 3–8 weeks in duration. For this study, “medical trainees” were defined collectively as medical students (undergraduate medical education, UME trainees) and residents and fellows (graduate medical education, GME trainees). Medical trainees were invited to participate via informational emails sent from study leads across nine participating institutions in the US between 2016 and 2020. Inclusion criteria are outlined in Figure 2. Participating institutions were members or affiliates of the Midwest Consortium of Global Child Health Educators [12]. A US$5 coffee card was offered as incentive for study completion. Previous pilot data confirmed that a sample size of at least 50 would produce a two‑sided 95% confidence interval of −0.829 to −0.499 for the effect of resilience on CSP score, assuming a correlation on −0.7.1 [3]. Consenting trainees were enrolled in REDCap (Research Electronic Data Capture*) [14, 15], which then autogenerated periodic surveys via Email based on the start date of their GH elective. Surveys included a predeparture survey (sent 7 days prior to the elective), on‑site periodic surveys (sent starting on day 2 of the elective and then every 5 days thereafter), and a postelective survey (30 days after return). Frequency was determined by pilot study participant feedback and aimed to increase likelihood of trainee completion of periodic assessments during the brief GH elective window. The data gathered with each survey are summarized in Figure 2.

### Primary outcome

Given the variable definitions of CS proposed in the literature, a consensus measurement tool for CS does not exist. St Clair et al. recently summarized tools that have been developed to measure CS [7]. Notably, only two offer opportunities for longitudinal assessments, and none have been previously utilized with medical providers. To measure our primary outcome (CS), we utilized Zapf’s “Culture Shock Profile” (CSP) 33‑point self‑assessment tool [16, 17]. This tool is composed of common symptoms experienced during cultural immersion, each graded on a scale of 0–3 and coded with a valence as positive or negative. The total CSP score can fall between 0 and 99, with higher scores representing greater CS. Originally developed to measure CS in social workers moving to isolated northern regions of Canada, the CSP tool was deemed internally consistent and feasible for use in medical trainees in a 2015 pilot study conducted by our team (Cronbach’s alpha 0.8–0.94) [13]. In this study, CSP scores were obtained at predeparture and sequentially thereafter (starting on day 2 of the elective and then every 5 days until completion of their GH elective).

### Secondary measures

**Figure 3:**
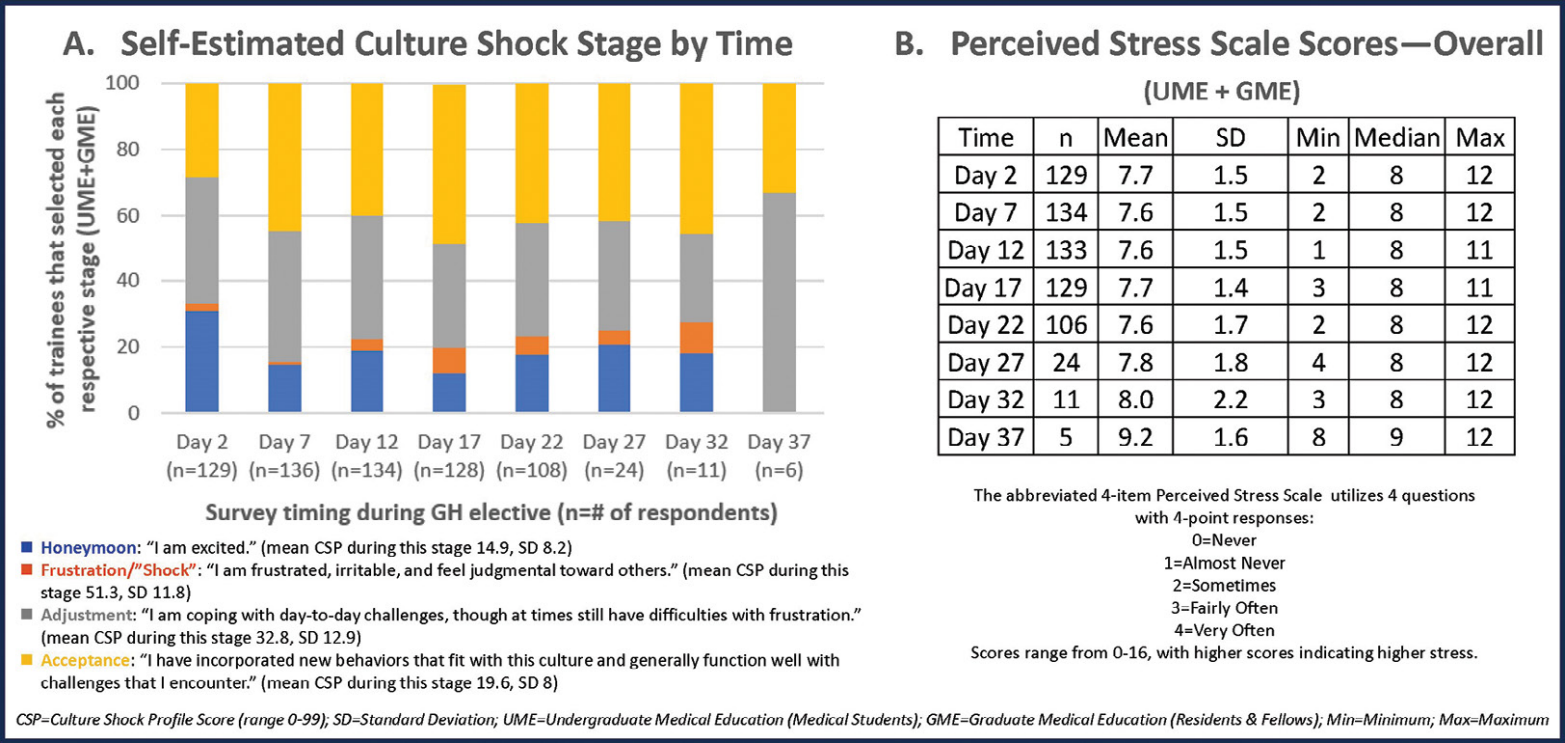
Summary of findings from the Culture Shock Stage Estimator (CSSE, **A**) and the Perceived Stress Scales (PSS) Scores (**B**).

Secondary measures were informed by a 2015 pilot study and literature review and included stress (abbreviated four‑item Perceived Stress Scale (PSS) [18]; Figure 3, B) and self‑estimated culture shock stage (CS Stage Estimator, CSSE; Figure 3, A). Given that there are no alternative CS measurement tools to assess convergence validity of the CSP, we chose the PSS to determine whether the CSP scores correlate with stress, acknowledging that stress is a limited and oversimplified proxy for CS. The CSSE was a new tool developed by the author team, not previously validated, created using the previously described stage‑based conceptual framework. Trainees were asked to choose which CS stage they felt most represented their current situation, and the stages were described as follows: Honeymoon: “I am excited”; frustration/“shock”: “I am frustrated, irritable, and feel judgmental toward others”; adjustment: “I am coping with day‑to‑day challenges, though at times still have difficulties with frustration”; and acceptance: “I have incorporated new behaviors that fit with this culture and generally function well with challenges that I encounter.”

### Covariates

Covariates collected in the predeparture survey and utilized in the model included: trainee demographic characteristics (gender, resilience score (Connor–Davidson resilience score 10 (CD‑RISC 10) [19]), trainee type, and language fluency), prior international work experience, GH preparation methods, and number of other trainees also traveling (solo versus group). Covariates collected in the on‑site survey assessed training conditions using a five‑point Likert scale, including: ease of communication with clinical supervisor, ease of communication with patients, resource availability, support network on‑site, medical needs in the community, clarity of role, and degree of conflict related to local medical practices. Additional covariates included trainee’s clinical role, trainee’s degree of personal responsibility in patient outcomes, and levels of patient acuity and mortality.

### Analyses

We conducted several analyses from our dataset, some of which were summarized at previous meetings [20–22]. In this manuscript, participant demographic, training, and elective characteristics as well as CSSE and postreturn survey data were summarized by descriptive statistics. Correlation analyses assessed the relationship between PSS and CSP scores. Simple linear regression evaluated the association between predeparture resilience and CSP scores at each time point. Additionally, a Fisher’s exact test investigated differences in trainee sense of preparedness pre‑ versus postreturn. A multifaceted approach was employed to provide robust insights into the trainees’ experiences and outcomes.

A linear mixed effects model (LMM) was used to examine longitudinal associations between selected covariates and CSP scores. Covariates demonstrating statistically significant associations with the primary outcome were candidates for inclusion in the multivariable model, as were those informed from prior published research regardless of statistical significance in this study. Diagnostics were conducted to assess collinearity and model stability, resulting in the removal of the following variables: helpfulness of clinical supervisor, helpfulness of nonsupervisory providers, and expectation of trainee skill matching actual trainee skill. The primary outcome, CSP score, underwent a log(*x* + 1) transformation due to non‑normality. The LMM used an autoregressive covariance structure, with the covariates as fixed effects, and a random intercept and random slope by participant. The least‑square means from the linear mixed model were used to assess trends over time in CSP scores. To assess the potential impact of missing data, we conducted a multiple imputation sensitivity analysis. A sensitivity analysis was conducted using chi‑squared tests to assess differences between respondents included in the analyses and those excluded due to insufficient data. Analyses were conducted in SASv.9.4. *P* values < 0.05 were considered statistically significant.

### Study logistics

Study leadership (and the associated REDCap database*) transitioned from the Medical College of Wisconsin to the University of Wisconsin in 2017 due to relocation of the Principal Investigator (PI, NSC). Institutional review board (IRB) exempt or expedited approval was obtained from each participating institution for the duration of the study. Financial support for the participant coffee gift cards was provided by the University of Wisconsin Department of Pediatrics Research and Development Committee. Notably, our study team shared this research protocol in 2016 with the Global Health Service Partnership (GHSP) upon their request. GHSP is a public–private collaboration between Seed Global Health, the US Peace Corps, and the US Presidents Emergency Plan for AIDS Relief. They modified the protocol to conduct a similar study on medical and nursing health professionals (*n* = 12) engaged in their long‑term international placements; the results were published in *Annals of Global Health* in 2021 [23].

## Results

### Study population

We initially intended to collect data up until 2022, but the study was truncated in 2020 due to halting of GH electives with the coronavirus disease 2019 (COVID‑19) pandemic. By 2020, 252 medical trainees were enrolled, participating in GH electives in 41 different countries. Demographics of the study participants are summarized in Table 1, notable for majority white (54%), female (55%), and GME trainees (72% residents, representing a variety of specialty training programs). A total of 47% reported prior experience providing medical care outside of the USA. Most trainees participated in GH electives in Africa (52%, Figure 2), with a median duration of 27 days (range 17–88). A total of 53% indicated that they were traveling with others (22% solo, 25% did not respond; type of traveling companion (faculty versus peer) was not queried), and 29% indicated that they were at least partially fluent in the predominant language at the GH elective site. Mean predeparture resilience (CD‑RISC 10) scores were 25 (range 15–30; max score of 30 with higher scores representing higher resilience). Completion rates of all surveys are summarized in Figure 2.

**Table 1:** Baseline participant demographic, training, and medical elective characteristics by total and analyzed samples.

	TOTAL SAMPLE *N* (COL %)	ANALYSIS SAMPLE *N* (COL %)	*P* VALUE^1^
Total, *n*	252	140	
**Demographic characteristics**			
Age			
Mean (range)	29 (24–40)	29 (25–36)	0.13
Race and ethnicity			
White, non‑Hispanic	136 (54)	99 (71)	0.58
Hispanic	5 (2)	3 (2)	
Black, non‑Hispanic	10 (4)	8 (6)	
Asian or Pacific Islander, non‑Hispanic	26 (10)	21 (15)	
Multiracial, non‑Hispanic	4 (2)	2 (1)	
Missing	71 (28)	7 (5)	
Gender			
Male	56 (22)	41 (30)	0.89
Female	137 (55)	99 (70)	
Missing	59 (23)	0 (0)	
Marital Status			
Single	104 (41)	75 (54)	0.94
Married/partnered	80 (32)	58 (41)	
Other	9 (4)	7 (5)	
Missing	59 (23)	0 (0)	
Prior work experience outside USA			
Yes	131 (52)	97 (69)	0.45
No	61 (24)	42 (30)	
Missing	60 (24)	1 (1)	
Prior travel experience in elective site			
Yes	39 (16)	28 (20)	0.91
No	154 (61)	112 (80)	
Missing	59 (23)	0 (0)	
Prior work experience in elective site			
Yes	25 (10)	18 (13)	0.95
No	168 (67)	122 (87)	
Missing	59 (23)	0 (0)	
Provided medical care outside USA			
Yes	119 (47)	90 (64)	0.28
No	73 (29)	50 (36)	
Missing	60 (24)	0 (0)	
Predeparture resilience score (CD RISC‑10)			
Mean (range)	25 (13–30)	25 (15–30)	0.79
**Training characteristics**			
Training institution			
Institution no. 1	13 (5)	6 (4)	0.08
Institution no. 2	2 (1)	1 (1)	
Institution no. 3	37 (15)	22 (16)	
Institution no. 4	39 (15)	29 (20)	
Institution no. 5	38 (15)	14 (10)	
Institution no. 6	65 (26)	33 (24)	
Institution no. 7	30 (12)	19 (14)	
Institution no. 8	17 (7)	10 (7)	
Institution no. 9	6 (2)	2 (1)	
Missing	5 (2)	4 (3)	
Type of trainee			
Medical student (UME trainee)	63 (25)	37 (26)	0.04
Resident (GME trainee)	181 (72)	102 (73)	
Fellow (subspecialty GME trainee)	8 (3)	1 (1)	
Year of training			
Medical student (UME trainee) Year 2	1 (0)	1 (1)	0.006
Medical student (UME trainee) Year 3	4 (2)	1 (1)	
Medical student (UME trainee) Year 4	51 (20)	37 (26)	
Resident, (GME trainee) postgraduate year 2	20 (8)	19 (13)	
Resident, (GME trainee) Postgraduate year 3	104 (41)	77 (55)	
Resident, (GME trainee) Postgraduate year 4	9 (4)	4 (3)	
Fellow (subspecialty GME trainee)	4 (2)	1 (1)	
Missing	59 (23)	0 (0)	
Completion of a simulation preparation activity			
Yes	129 (51)	96 (69)	0.41
No	64 (26)	44 (31)	
Missing	59 (23)	0 (0)	
Predeparture meeting(s) with a faculty mentor			
Yes	172 (68)	129 (92)	0.03
No	21 (9)	11 (8)	
Missing	59 (23)	0 (0)	
Predeparture curriculum			
Yes	178 (71)	128 (91)	0.76
No	15 (6)	12 (9)	
Missing	59 (23)	0 (0)	
Didactic overview or independent reading about culture shock			
Yes	164 (65)	122 (87)	0.17
No	29 (12)	18 (13)	
Missing	59 (23)	0 (0)	
Predeparture online module(s)			
Yes	124 (49)	92 (66)	0.45
No	68 (27)	47 (33)	
Missing	60 (24)	1 (1)	
Discussions with trainees who worked at same or similar GH elective site			
Yes	155 (61)	117 (83)	0.05
No	37 (15)	22 (16)	
Missing	60 (24)	1 (1)	
Researching the country of the GH elective			
Yes	172 (68)	125 (89)	0.90
No	21 (9)	15 (11)	
Missing	59 (23)	0 (0)	
Language study/course in the host language			
Yes	42 (17)	35 (25)	0.08
No	151 (60)	105 (75)	
Missing	59 (23)	0 (0)	
**Medical elective characteristics**			
Region			
Africa	131 (52)	63 (45)	0.19
Asia	57 (23)	37 (26)	
Caribbean	10 (4)	7 (5)	
Central America	21 (9)	14 (10)	
Europe	10 (4)	5 (4)	
North America	11 (4)	5 (4)	
Oceania	0 (0)	0 (0)	
South America	11 (4)	8 (5)	
Missing	1 (0)	1 (1)	
Duration, days			
Median (Range)	27 (17–88)	26 (17–54)	0.34
Travel Cohort			
Solo	55 (22)	39 (28)	0.58
Group	135 (53)	101 (72)	
Missing	62 (25)	0 (0)	
Elective site language ability			
Not fluent	118 (47)	82 (59)	0.13
Partially, conversationally, and/or medically fluent	73 (29)	58 (41)	
Missing	61 (24)	0 (0)	

^1^ Differences between respondents analyzed versus excluded due to insufficient responses were assessed using *t*‑tests, chi‑square tests, and Fisher’s exact tests as appropriate.

### CSP as an assessment tool for CS

Because our primary outcome was the CSP score (ranging from 0 to 99), and the CSP self‑assessment tool has not been previously used in large scale studies nor in medical providers, we assessed internal consistency and convergence validity. It demonstrated excellent internal consistency with each survey time point (Cronbach’s alpha > 0.9). Regarding convergence validity, we did not find a significant linear relationship between the CSP and PSS scales. The CSSE, our assessment tool that asked trainees to self‑identify their current stage of CS, potentially provided some validation of the CSP tool, demonstrating expected low mean CSP scores during the honeymoon stage (14.9, standard deviation (SD 8.2) and high during the frustration/shock stage (51.3, SD 11.8). Mean CSP scores for the adjustment and acceptance stages were 32.8 (SD 12.9) and 19.6 (SD 8), respectively. PSS scores and CSSE results are summarized in Figure 3, A and B.

### Influence of predeparture resilience on CSP scores

Our 2015 pilot study suggested that predeparture resilience inversely correlates with CSP scores. A simple linear regression conducted at each survey point in this study again demonstrated statistically significant inverse relationships between resilience and the CSP estimate at the following points: predeparture (coefficient −0.66), on‑site day 2 (−0.7), day 12 (−0.96), day 17 (−0.86), day 27 (−2.54), and day 32 (−3.31). However, when adjusting for all variables, predeparture resilience did not significantly attenuate CSP scores (Table 2).

**Table 2:** Adjusted and unadjusted associations between selected predictors and Culture Shock Profile (CSP) score using a linear mixed effects model, *n* = 140^1^.

	ADJUSTED^2^				UNADJUSTED			
	ESTIMATE	PERCENT CHANGE^3^	SE	*P* VALUE	ESTIMATE	PERCENT CHANGE	SE	*P* VALUE
**INTRINSIC FACTORS (DEMOGRAPHICS)**								
Resilience score (CD RISC‑10)	−0.0126	−1.25	0.01	0.2078	−0.03	−3.31	0.01	0.0009*
Male gender (reference: female gender)	−0.0536	−5.22	0.07	0.4638	0.10	10.97	0.08	0.1979
Medical student, UME trainees (reference: resident and fellow, GME trainees)	0.1971	21.79	0.09	0.0385*	0.07	6.79	0.08	0.4238
Traveled alone (reference: traveled with others)	0.0899	9.41	0.08	0.2379	0.00	−0.06	0.08	0.9941
Prior provision of medical care outside USA (reference: no prior experience)	−0.0163	−1.62	0.07	0.8159	−0.14	−12.67	0.07	0.0472*
Completion of simulation for a preparation activity (reference: no simulation activity)	0.0831	8.67	0.09	0.3352	0.02	1.99	0.08	0.7969
Partially, conversationally, and/or medically fluent (reference: not fluent)	0.0321	3.26	0.08	0.6765	−0.25	−22.41	0.08	0.001*
**EXTRINSIC FACTORS (TRAINING CONDITIONS)**								
“I think there is a good support network here to help me deal with difficult situations” (support network on‑site)	−0.1053	−9.99	0.03	<0.0001*	−0.19	−17.40	0.02	<0.0001*
“I can communicate easily with my clinical supervisor and non‑supervisory providers” (ease of communication with clinical supervisor and non‑supervisory providers)	−0.0701	−6.77	0.02	0.0036*	−0.13	−12.22	0.02	<0.0001*
“I can communicate easily with my patients” (ease of communication with patients)	−0.0684	−6.61	0.03	0.0084*	−0.13	−11.90	0.02	<0.0001*
“There are sufficient resources to provide good care for my patients” (resource availability)	0.0033	0.33	0.03	0.8996	−0.08	−7.87	0.02	0.0002*
“I feel overwhelmed by the medical needs in this community” (medical needs in the community)	0.0939	9.84	0.03	0.0005*	0.11	12.00	0.02	<0.0001*
“I have a clear sense of my role and how I can be helpful” (clarity of role)	−0.1175	−11.09	0.02	<0.0001*	−0.18	−16.20	0.02	<.0001*
“I feel conflicted between my own ideas of medical care and a desire to respect local medical practices that differ” (degree of conflict related to local medical practices)	0.0592	6.10	0.02	0.0103*	0.09	9.74	0.02	<0.0001*
“Number of patient deaths that have occurred over the past 5 days that I am aware of” (patient mortality)	0.0186	1.88	0.01	0.0279*	0.03	2.69	0.01	0.0007*
“Increasing severity of health status of the majority of patients over the past 5 days” (level of patient acuity)	0.0304	3.09	0.03	0.3508	0.07	7.11	0.03	0.0250*
“My role during the GH elective is a clinical provider” (reference: observer or educator)	0.0340	3.46	0.06	0.6021	−0.07	−6.34	0.05	0.2268
“Percent of personal responsibility that I felt for the outcomes of my patients over the past 5 days”^4^	−0.0003	−0.03	0.00	0.7895	0.00	−0.12	0.00	0.2456

Notes: SE, Standard Error.

^1^ Statistically significant difference over time determined by linear mixed effects methods with a random intercept and slope, where the continuous outcome underwent a log(*x* + 1) transformation, predictors were modeled as categorical or continuous variables, and time modeled as a categorical variable.

^2^ The adjusted model includes all covariates. The unadjusted model examines each variable independently.

^3^ To ease interpretation, the percent change indicated by the slope estimate via standard back‑transformation is provided. For example, for every one‑unit increase in the independent variable (resilience score), our dependent variable (Culture Shock Profile score) decreases by a factor of about 0.0126 or 1.25%.

^4^ Percent of personal responsibility is operationalized as 0% meaning “you did not feel responsible for patient outcomes,” 50% meaning “you shared responsibility equally with other providers,” and 100% meaning “you felt fully responsible for patient outcomes”.

*Denotes significant *P* value < 0.05.

### Influence of predeparture preparation on CSP scores

A total of 92% of participants reported completion of some form of predeparture curriculum at their institution. Before departure, 87% reported feeling “somewhat or very prepared.” There was not a statistically significant change in self‑perceived preparedness post return (90% reported feeling “somewhat or very prepared” post return, *P* = 0.54). When using participation in predeparture simulation activities as a proxy for preparedness, our multivariable (LMM) analysis did not demonstrate significant attenuation in CSP scores related to preparedness.

### Patterns of CS experienced by medical trainees during GH electives

**Figure 4:**
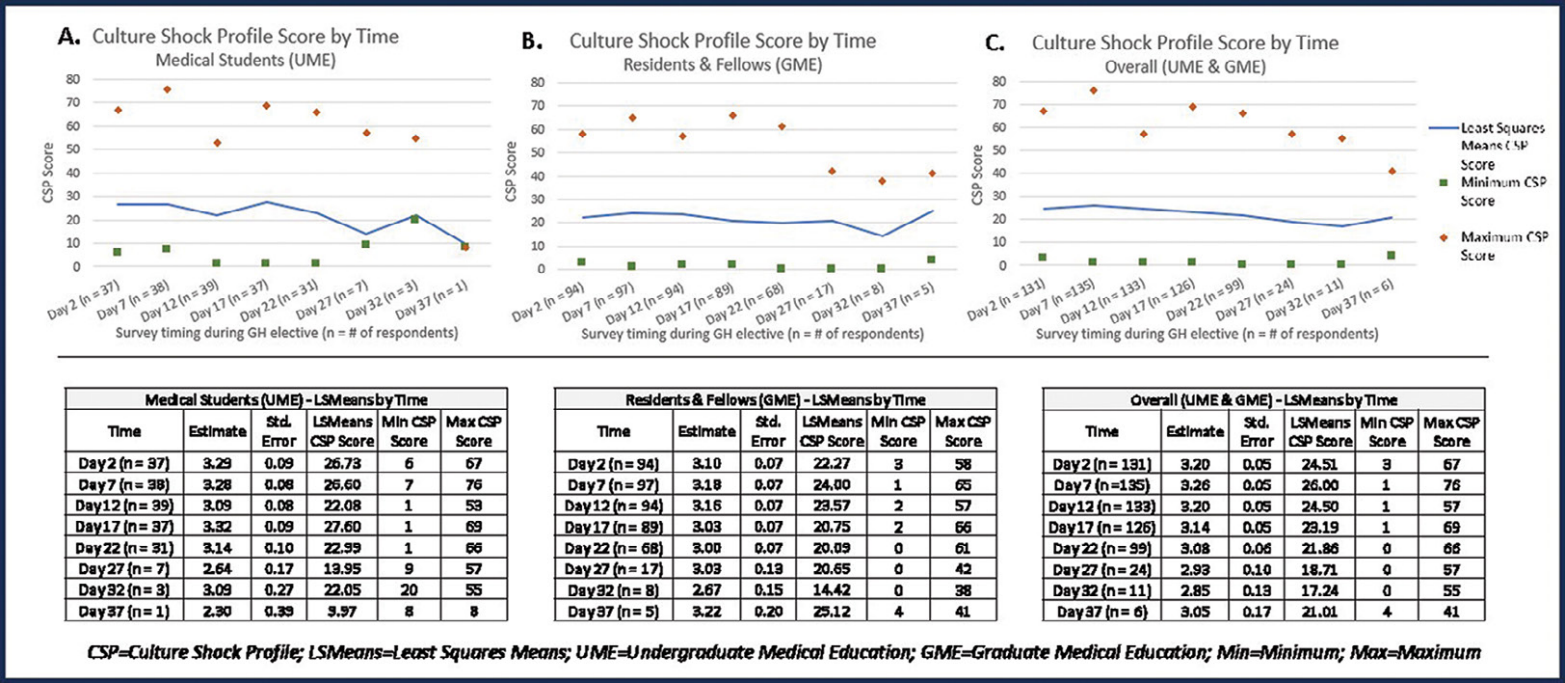
Mean CSP scores over time for (**A**) medical students; (**B**) residents and fellows; and (**C**) medical students, residents, and fellows combined.

As a population, medical trainee CSP least square mean scores fluctuated between 17 and 26 over the course of GH electives, with large individual ranges of scores across all survey time points (Figure 4, A–C). As shown in the CSP curves, there was not a predictable “peak” in CSP scores at a particular time point during the electives. Similarly, the CSSE revealed that trainees, as a population, do not progress through the various stages of CS in a strictly time‑dependent nor linear manner (Figure 3, A). “Frustration/shock” was the least frequently self‑identified stage in the Culture Shock Stage Estimator across all time points, while “adjustment” and “acceptance” were consistently the most frequently chosen stages. Of note, we attempted to better understand the factors that correlated with CSP scores ≥50 (the mean score noted during the “frustration/shock” phase was 51.6) but were unable to run the multivariable analysis due to insufficient time points with CSP scores ≥50.

### Postreturn medical trainee perceptions of CS

From the postreturn survey, CS appeared to be a near‑universal experience for the study participants: 96% reported experiencing CS during and 74% after their GH electives (reverse/reentry CS), with 53% stating that they were impacted somewhat or very significantly during and 32% after (Table 3). Trainees indicated that they felt CS negatively impacted these factors at the following frequencies during the GH elective: overall mood (31%), opinions about the local clinical environment (30%), interactions with local providers and patients (15%), ability to perform activities of daily living such as sleeping and eating (14%), desire to continue participating in the GH elective (14%), clinical performance (14%), and interactions with local community members (13%). Post return, trainees stated that reverse CS had a negative impact on the following factors: their perceptions of how medicine is practiced at their home institutions (35%), overall mood (22%), their ability to communicate easily with family and friends (18%), and their ability to perform normal life functions (11%) [20].

**Table 3:** Impact of culture shock and reverse culture shock on medical trainees during and after global health electives (data obtained from postreturn survey of medical students, residents, and fellows (UME and GME trainees), *n* = 147^1^).

**How would you describe your experience of…**									
	Not significant *n* (row %)		Minimally significant *n* (row %)		Somewhat significant *n* (row %)		Very significant *n* (row %)		
Culture shock during your GH elective?	6 (4)		63 (43)		66 (45)		11 (8)		
REVERSE culture shock after your return?	38 (26)		60 (42)		38 (26)		8 (6)		
**The experience of culture shock had a NEGATIVE impact on my…**									
	Strongly disagree *n* (row %)		Disagree *n* (row %)		Neutral *n* (row %)		Agree *n* (row %)		Strongly agree *n* (row %)
Ability to work with other local providers	38 (26)		66 (46)		17 (12)		22 (15)		1 (1)
Interactions with patients	35 (25)		69 (49)		16 (11)		22 (15)		0
Interactions with local community members	35 (25)		67 (47)		21 (15)		19 (13)		0
Ability to perform normal life functions (sleeping, eating, etc.)	48 (33)		57 (40)		19 (13)		19 (13)		1 (1)
Clinical performance	31 (22)		65 (46)		26 (18)		19 (14)		0
Overall mood	30 (21)		45 (31)		25 (17)		37 (26)		7 (5)
Opinions about the local clinical environment	27 (19)		52 (37)		20 (14)		41 (29)		2 (1)
Desire to continue the elective	60 (42)		52 (36)		12 (8)		16 (11)		4 (3)
**The experience of REVERSE culture shock had a NEGATIVE impact on my…**									
	Strongly disagree *n* (row %)		Disagree *n* (row %)		Neutral *n* (row %)		Agree *n* (row %)		Strongly agree *n* (row %)
Ability to communicate easily with friends and family after my return	50 (35)		51 (35)		17 (12)		25 (17)		1 (1)
Ability to perform normal life functions (sleeping, eating, etc.)	50 (35)		65 (45)		12 (8)		17 (12)		0
Overall mood	48 (34)		49 (34)		15 (11)		29 (20)		2 (1)
Perceptions on how medicine is practiced at my home institution	27 (19)		40 (28)		26 (18)		41 (28)		10 (7)

^1^ Missing data is excluded from this analysis.

### Findings from the multivariable analysis

We utilized a linear mixed effects model (LMM) to conduct a multivariable analysis of our dataset, summarized in Table 2. Of the 252 trainees, the following participants were removed from the dataset for the purposes of this analysis: 4 (nonparticipation in an elective), 56 (noncompletion of the predeparture survey), and 52 (missing covariates that precluded utilization in the LMM, resulting in a total of 140 medical trainees analyzed in the LMM). We conducted model diagnostics and removed three variables from the model due to collinearity.

The intrinsic factors describe the variables inherent to the trainees (gender, prior experience, language fluency, etc.). Of those, the only variable that significantly influenced CSP scores was the type of trainee, with medical students (UME trainees) demonstrating 22% higher CSP scores compared with residents and fellows (GME trainees). The extrinsic factors summarize the variables related to training conditions at the GH elective site (support network, ease of communication, patient acuity, etc.). Of those, multiple variables significantly influenced CSP scores including: support network on site (−10% change in CSP score over all time points), ease of communication with clinical supervisor and patients (−7%), sense of feeling overwhelmed by medical needs in the community (+10%), clarity of professional role (−11%), sense of conflict between one’s own ideas of medical care and a desire to respect local practices that differ (+6%), and patient deaths (+2%).

Our dataset had approximately 20% missing values, related to incomplete or skipped surveys. To assess the potential impact of missing data, we conducted a multiple imputation sensitivity analysis, which revealed slight differences in the results from those outlined in Table 2. Specifically, the following variables no longer showed a significant association with CSP scores: trainee type (*P* = 0.19) and ease of communication with clinical supervisor (*P* = 0.14) or with patients (*P* = 0.11).

## Discussion

We sought to quantitatively measure medical trainee psychosocial responses to short‑term GH training experiences, to identify factors that influence the severity of CS experienced by traveling trainees and to determine whether medical trainees navigate through CS in a stage‑based fashion. In our population, almost all trainees reported experiencing CS during their GH electives, though the severity of their CSP scores (as a proxy measurement for CS) and perceived personal and professional impact of CS were highly variable. Extrinsic factors (training conditions) influenced the trainee’s CS experience more than intrinsic factors (demographics). Furthermore, their emotional responses to cross‑cultural immersion did not progress in the predictable, curvilinear, stage‑based fashion previously described in literature (Figure 1) [8, 9].

Medical trainees rarely self‑identified as being in the “frustration/shock” phase. In addition, despite a wide range of CSP scores for each periodic assessment during the GH electives, the overall average scores were not high and did not vary markedly throughout the elective, particularly for GME trainees. These mostly temperate average CSP scores for the study population, paired with the high pre/post rates of perceived preparedness, speaks to a highly prepared and resilient cohort of medical trainees, as well as supportive learning environments. However, despite the overall population trends depicting an unstressed cohort of traveling medical trainees, our data suggest that a minority of them were significantly affected by cross‑cultural immersion stress: CSP score ranges varied widely, and 20–30% reported that their CS experience negatively impacted their professional performance or personal well‑being during and after the elective. These findings indicate that CS remains an important consideration for medical trainees and their hosts during GH electives.

As mentioned, factors that significantly influenced CSP scores were related to the on‑site training conditions and trainee type but not other measured trainee‑specific factors (such as gender, resilience, preparation methods, travel companions, and prior travel experiences). Trainee characteristics that were not measured in this study (e.g., social emotional health, empathy, mindfulness, pre‑existing mental health conditions, individual biases, etc.) [24] undoubtedly influenced CSP scores and CS experiences in variable ways. However, the fact that our results did not identify a vulnerable traveler profile (e.g., a set of demographic characteristics that consistently correlated with higher CSP scores) and instead suggested that CS experiences are influenced more by on‑site conditions (many of which are modifiable), highlights the importance of optimizing global training partnerships and associated training scaffolding.

The diverse patterns of CS noted in this study population, both in severity (assessed by CSP scores) and stage (assessed by the CSSE) suggests that the stage‑based conceptual framework, while helpful in offering an explanation of potential emotional responses during cross‑cultural immersions, may not apply to medical trainees given the complexities of short‑term GH training, the potential mitigating impact of pre‑travel preparation, and perhaps unique characteristics inherent to medical trainees who choose to pursue GH electives.

Project PRIME’s results have notable implications for medical trainees, educators, and international hosts. Historically, predeparture training before GH electives emphasized the stage‑based CS experience and focused primarily on trainee‑specific strategies to mitigate the potential negative effects of CS. Such strategies include predeparture, on‑site, and postreturn reflection; regular communication with home supports and faculty mentors; development of an on‑site support network; pursuit of a sense of purpose during the GH elective; and self‑compassion [7, 25–30]. Our findings suggest that, while these strategies are important, preparation practices should also include: (1) avoiding expectations of stage‑based, predictable CS experiences, and instead acknowledging that individual experiences may be highly variable; (2) reassuring trainees that well‑being is commonly maintained despite experiences of CS, while still acknowledging that a minority may experience significant personal and professional negative impacts; (3) fostering direct communication and goal expectation between the trainee and international host(s) prior to departure; (4) establishing on‑site communication plans prior to departure (with both home mentors and on‑site hosts); (5) collaborating with international hosts to optimize extrinsically modifiable variables at the elective site that significantly correlate with CSP scores (Table 2); and (6) improving the GH educator community’s infrastructure to standardize and operationalize transparent evaluations soliciting trainee, stateside mentor, and international host perspectives for the purposes of GH elective and GH partnership quality improvement. Additionally, given the clear influence of on‑site training conditions, our findings reiterate the importance of collaborating with international hosts through sustained, mutually beneficial global training partnerships, supporting ongoing communication, and providing resources and compensation for their work related to hosting visiting trainees [31–33].

## Limitations

Our study had several limitations. It is important to note that these medical trainees were not just experiencing psychosocial responses to cross‑cultural immersion, but also to professional immersion in distinctly different clinical settings. It was impossible to differentiate between CS and “professional shock” in this study (e.g., psychosocial responses to working with resource limitations, unknown diagnoses, and high patient acuity); a mixed methods approach potentially could have assisted in differentiating between the two. We additionally were unable to assess through this study design and population: (1) trainee‑specific Cultural Distance Index [34], (2) influence of the duration of GH electives, (3) impact of trainee‑specific personality traits and social emotional health, (4) year‑to‑year variation, (5) qualitative assessments of the trainee and host experiences, (6) impact of traveling with a stateside faculty mentor, and (7) reasons for and implications of missing data.

Our primary outcome, CSP, demonstrated excellent internal consistency as an assessment tool for CS but would benefit from further validation in future studies. Such investigations could also include the creation of graded scoring to differentiate between “mild, moderate, and severe” CS, which we were unable to do with our study design.

Our study may have been impacted by selection bias when compared with a national sample; specifically, our participants were at Midwestern institutions with robust GH training infrastructures, and most participants had previously traveled internationally, had internet access during their GH electives, and reported high rates of predeparture preparation. Interestingly, the high rates of predeparture preparation in our cohort may have accounted for our team not finding a significant correlation between preparation and CSP scores (as compared with the 2021 Global Health Service Partnership study, which found that low levels of predeparture preparedness correlated with high levels of CS) [23]. Participants were also vulnerable to social desirability bias (i.e., participants knew that their responses would be visible to the study PI and linked with their identity due to use of personal e‑mails for survey distribution).

This study was abruptly halted by the COVID‑19 global pandemic. While we gathered sufficient data to achieve statistical significance for our prepandemic study findings, our world has since significantly changed, including attitudes about and practices pertaining to GH electives. Medical trainee CS and GH elective experiences may therefore differ in future years.

Finally, and most importantly, due to feasibility barriers, international host perspectives were not represented in the study design, analysis, nor interpretation of these findings. Little is known about host experiences related to medical trainee CS, and host input would have enriched both the development of the study protocol and the interpretation of our findings. An essential follow‑up project will be to study international host experiences, obtain reflection on the impact of CS experiences on host communities, and solicit suggestions on methods to optimize the modifiable variables that were found to significantly impact CS in our study.

## Conclusion

CS is a near‑universal experience for US medical trainees during GH electives. We know that some CS is potentially beneficial—promoting transformative learning, professional identity formation, cultural humility, and resilience [4–7]. However, at the extremes, CS is potentially harmful—impairing trainee well‑being and negatively impacting host communities [10]. It is, therefore, imperative that our medical education community better understand CS to support the well‑being of our traveling medical trainees and international hosts. This study suggests that medical trainee CS experiences are highly variable and primarily influenced by on‑site training conditions, many of which are modifiable through sustained global training partnerships. Further research is required to (1) determine the optimal CS “balance” for traveling trainees (one that promotes transformative learning while mitigating negative professional and personal impacts), (2) offer insight into harmful CS thresholds, (3) identify host perspectives related to the CS experience for visiting medical trainees, and (4) inform best practices for GH electives and global training partnerships.

## Abbreviations

GH: Global Health

CS: Culture Shock

CSP: Culture Shock Profile

CSSE: Culture Shock Stage Estimator

CD‑RISC 10: Connor Davidson Resilience Scale 10

GME: Graduate Medical Education

LSMeans: Least Squares Means

LMM: Linear Mixed Effects Model

PI: Principal Investigator

PSS: Perceived Stress Scale

REDCap: Research Electronic Data Capture

UME: Undergraduate Medical Education

US: United States
